# Antibody Response After a Fifth Dose (Third Booster) of BNT162b2 mRNA COVID-19 Vaccine in Healthcare Workers

**DOI:** 10.3390/jcm13216538

**Published:** 2024-10-31

**Authors:** Esther Saiag, Ronni Gamzu, Hagit Padova, Yael Paran, Ilana Goldiner, Neta Cohen, David Bomze

**Affiliations:** 1 Division of Information Systems and Operations, Tel Aviv Sourasky Medical Center, Tel Aviv 6423906, Israel; esthers@tlvmc.gov.il; 2 Sackler Faculty of Medicine, Tel Aviv University, Tel Aviv 6997801, Israelnetaco@tlvmc.gov.il (N.C.); 3 Tel Aviv Sourasky Medical Center, Tel Aviv 6423906, Israel; 4 Department of Quality and Patient Safety, Tel Aviv Sourasky Medical Center, Tel Aviv 6423906, Israel; 5 Department of Infectious Diseases and Infection Control, Tel Aviv Sourasky Medical Center, Tel Aviv 6423906, Israel; 6 Division of Clinical Laboratories, Tel Aviv Sourasky Medical Center, Tel Aviv 6423906, Israel; 7 Emergency Department, Tel Aviv Sourasky Medical Center, Tel Aviv 6423906, Israel; 8 Division of Dermatology, Tel Aviv Sourasky Medical Center, Tel Aviv 6423906, Israel

**Keywords:** booster, vaccine, SARS-CoV-2, COVID-19, BNT162b2

## Abstract

Although a fourth dose of SARS-CoV-2 vaccine was shown to be effective, the immunogenicity of a fifth dose in immunocompetent individuals had not been well described. This was a prospective observational cohort study of previously vaccinated healthcare workers at a single tertiary hospital in Israel. Individuals were administered up to three booster doses of the BNT162b2 mRNA vaccine (i.e., up to five overall doses), during the period between July 2021 and January 2023. Immunogenicity was assessed using the SARS-CoV-2 IgG (sCOVG) semi-quantitative assay, performed at several time points. The cohort consisted of 162 individuals (median age 69 years, 62% female). Of these, 104 (64%) received four doses and 58 (36%) received five doses. Anti-SARS-CoV-2 antibody levels increased in all cases, regardless of the baseline levels. The fold-change increase in the mean sCOVG index was 29.2 (SD 2.6) after the third vaccine, 3.8 (SD 2.4) after the fourth vaccine, and 3.6 (SD 3.0) after the fifth vaccine. A waning effect over time was seen in 78% and 43% of participants for the third and fourth doses, respectively. Adverse events following the fifth dose were limited and mild. Similar to previous booster vaccines, a fifth dose of BNT162b2 is immunogenic and safe in healthy individuals, although the clinical implications remain unclear.

## 1. Introduction

The COVID-19 pandemic is a defining event in modern history. The disease, whose long-term consequences are still unknown, has already claimed the lives of millions. Despite the success of early vaccination efforts, the advent of the deadly B.1.617.2 (delta) variant resulted in the rollout of a third booster dose during late 2021, which was shown to be immunogenic and effective [1,2,3,4].

In early 2022, the emergence of the B.1.1.529 (omicron) variant and the subsequent upsurge in cases led the Israeli government to offer a fourth vaccine dose to healthcare workers and people older than 60 years of age. Individuals immunized with four doses had a substantial risk reduction for COVID-19-related hospitalizations and deaths compared to those who received three doses [5]. The emergence of a new virulent strain, which may require an additional booster in selected populations, is not beyond imagination. Fortunately, any ancestral-based (and not only variant-modified) vaccine is expected to boost anti-SARS-CoV-2 immunity [6]. However, little is known about the real-world safety and immunogenicity of a fifth dose. Here, we report the effect of multiple COVID-19 vaccine boosters, up to and including a fifth dose, in healthcare workers.

## 2. Materials and Methods

We conducted a prospective, single-center observational study to investigate the effect of multiple COVID-19 booster doses. The cohort consisted of Tel Aviv Sourasky Medical Center (TLVMC) healthcare workers aged 60 years or older. All individuals had received two doses of the BNT162b2 vaccine (30 mcg in 0.3 mL delivered to the deltoid muscle) in late 2020, a third dose (i.e., first booster) in August 2021, a fourth dose (i.e., second booster) in January 2022, and some were given a fifth dose (i.e., third booster) in the period between November 2022 and January 2023.

The presence of neutralizing IgG antibodies against the receptor-binding domain (RBD) of the SARS-CoV-2 spike protein S1 subunit was evaluated via a chemiluminescent microparticle immunoassay on the ADVIA Centaur XP System (Siemens, Tarrytown, NY, USA). This semi-quantitative SARS-CoV-2 IgG assay provides an index value (termed sCOVG) between 0 and 100.00. An index greater than 1.00 is considered reactive for SARS-CoV-2 IgG antibodies. According to the established World Health Organization (WHO) international standard, an sCOVG index value of 1.00 corresponds to a binding antibody unit per milliliter (BAU/mL) value of 21.8 [7]. Antibody levels were summarized using the geometric mean. The fold-change between the pre- and post-vaccine levels was calculated by taking into account only individuals with pre-vaccine levels less than 100.00 (the upper limit of the measuring interval). Adverse events were recorded using a structured questionnaire sent to participants 3–7 days after the fifth vaccine.

All statistical analyses were performed in R, version 3.5.0. This study was approved by the TLVMC institutional review board (approval #TLV-21-0587).

## 3. Results

A total of 162 healthcare workers received at least four BNT162b2 doses and were included in the analysis. Of these patients, 58 (36%) received five vaccine doses (Table 1), and 104 (64%) received four vaccine doses. The median age was 69 years (interquartile range [IQR], 65–74 years), and 62% were female. Although participants were considered mostly healthy, 61% self-reported some chronic medical condition, including dyslipidemia (46%), hypertension (33%), diabetes (14%), ischemic heart disease (7%), history of cancer (6%), and rheumatologic disease (3%). The median time between the third and fourth vaccine dose was 22 weeks and 44 weeks between the fourth and fifth dose. Individuals had their antibody levels measured the day the vaccines where given (immediately prior to administration), and some were additionally tested during various time points—one week and fifteen weeks after the third dose, three weeks after the fourth dose, and three weeks after the fifth dose. Overall, 23% (37/162), 23% (38/162), 41% (66/162), 3% (5/162), and 10% (16/162) had two, four, five, six, and seven available measurements, respectively.

**Figure 1:**
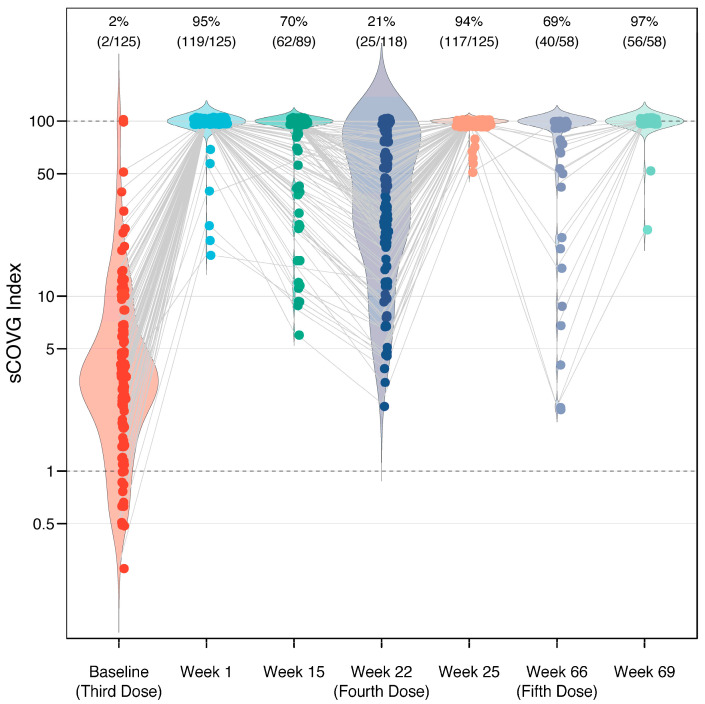
Immunogenicity of a third, fourth, and fifth dose of BNT162b2 vaccine in 162 healthcare workers. Response measured using a semi-quantitative serological assay, which reports a value (sCOVG index) up to 100.00, where an index ≥1.00 is considered positive for SARS-CoV-2 antibodies. Each dot represents one case, with gray lines connecting the same individual across longitudinal measurements. The width of the violin plot represents the distribution of the data along the *y*-axis. Following the third, fourth, and fifth doses, the sCOVG index remained >100.00 (the upper limit of quantification) in 95%, 94%, and 97% of individuals, respectively (labeled on top). The baseline levels prior to each dose display a gradual increase.

**Figure 2:**
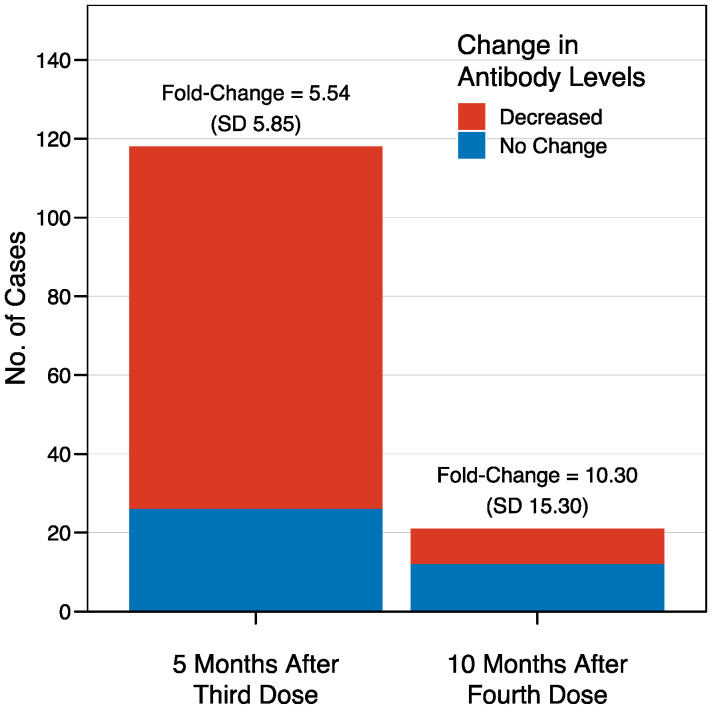
Waning immunity after the third and fourth doses of BNT162b2 vaccine in healthcare workers. Five months after the third dose (left), the antibody levels were decreased in 92 cases out of the 118 for whom longitudinal data were available, corresponding to 78%, with a mean fold-change sCOVG index decrease of 5.54 (SD 5.85). The decrease was less evident ten months after the fourth dose (right), with lower antibody levels seen in only 9 cases out of the 21 with available data (43%), but with a stronger effect size, reflected by a fold-change decrease of 10.30 (SD 15.30).

The mean sCOVG index at the baseline was 3.4 (standard deviation [SD] 2.9), and increased to >100.00 (the upper limit of quantification) in 95% of the recipients after the third dose, in 94% of the recipients after the fourth dose, and in 97% of the recipients after the fifth dose (Figure 1). This corresponded to a fold-change in the geometric mean of 29.2 (SD 2.6), 3.8 (SD 2.4), and 3.6 (SD 3.0) after the third, fourth, and fifth doses, respectively. There were no recipients who were non-reactive (sCOVG < 1.00) after any of the boosters. A waning in immunity was observed in individuals for whom longitudinal data were available (Figure 2). Antibody levels decreased in 78% of patients (92/118 cases) in the period between the third and fourth dose (over five months). Interestingly, the effect was less pronounced in the period between the fourth and fifth dose, with decreased antibody levels seen in only 43% of patients (9/21 cases with available data) over ten months. In contrast, the effect size was stronger in the latter period, with a mean fold-change sCOVG index decrease of 10.30 (SD 15.30), compared to the former period, with a fold-change decrease of 5.54 (SD 5.85). However, drawing conclusions from this comparison is difficult due to the different time span.

The fifth dose demonstrated a very good safety profile (Table 2). Early adverse events included mild local pain (55%), local erythema (16%), swelling (10%), and movement-restricting pain (6%). A total of 71% of cases had no symptoms after 48 h, whereas 18% reported fatigue, 8% reported myalgia or arthralgia, 6% reported fever, and 6% reported headaches. When asked to compare their experience with previous immunizations, 88% of individuals stated that the symptoms after the fifth dose were similar or milder, and only 6% stated that their symptoms were more severe. No serious adverse events were reported by any of the study participants.

## 4. Discussion

Despite the evidence gap surrounding a fifth mRNA COVID-19 vaccine dose in healthy populations, which this work aims to address, several studies suggest a benefit of a fifth dose in high-risk populations, including people living with HIV and organ transplant recipients [8,9,10]. Interestingly, a recent analysis of Japanese dialysis patients observed an increased proportion of cases who did not develop cellular immunity after a fifth dose, suggesting possible T-cell exhaustion and immune tolerance following repeated vaccinations [11]. An animal study that used repeated dosing of recombinant receptor-binding domain (RBD) booster vaccines in BALB/c mice might offer a mechanistic explanation for the above phenomenon [12]. This work showed that, once a vaccine response was established, extended immunization led to impaired CD4+ and CD8+ T-cell activity, the upregulation of immune checkpoints, an increased proportion of regulatory T-cells, and elevated levels of IL-10—a critical anti-inflammatory cytokine. In contrast, repeated ex vivo exposure of human CD8+ T-cells to SARS-CoV-2 antigen maintained the diversity of the T-cell receptor (TCR) repertoire and did not exhaust T-cells, indicating the efficacy and utility of booster vaccines, if needed [13].

Previous works in healthy individuals showed that a fourth dose boosts cellular and humoral immunity [14,15]. Regev-Yochay et al. reported that a fourth dose led to a 9- to 10-fold increase in titers of anti-SARS-CoV-2 IgG antibodies and neutralizing antibodies, an effect slightly stronger than the one achieved after a third dose [16]. A nationwide study of the Israeli population showed a decrease in the short-term risk of COVID-19-related outcomes among recipients of four vaccine doses, with relative effectiveness of 45% against confirmed infection, 62% against severe disease, and 74% against COVID-19 mortality [17]. Another analysis of the national Israeli Ministry of Health national database consistently showed that a fourth dose reduced the rates of both SARS-CoV-2 confirmed infection and severe COVID-19 illness, although protection against the former waned quickly [18,19]. According to another prospective study, the additional immunologic advantage of a fourth BNT162b2 vaccine was much smaller compared to a third dose, and waned completely within months, proposing the appropriate timing of booster doses to coincide with disease waves [20].

Two recent modeling studies on simulated endemic populations predicted the immuno-protection of a fifth vaccine dose [21,22]. Our work, using real-world data, does in fact show that a fifth dose resulted in a consistent serological response in all individuals. This finding is of particular importance given the waning immunity observed months after the administration of boosters. Whether this translates into a clinical effect in terms of infectivity, morbidity, or mortality is still an open question. A prospective cohort study in long-term care facilities in England (VIVALDI) showed that successive booster doses provide short-term protection against SARS-CoV-2-related mortality, but there was no long-term benefit from fourth- or fifth-dose vaccination relative to the third booster dose [23]. However, one can assume that the immunogenic potential of a fifth dose, reported herein, is a pre-requisite for any relevant clinical outcomes.

Another important issue is the effect of “hybrid immunity” resulting from the combination of natural SARS-CoV-2 immunity and vaccine-generated immunity, which may lead to a synergistic immune response [24]. A recent Japanese study reported that high antibody titers, equivalent to those seen immediately after the second vaccination, are maintained more than one year after COVID-19 infection in previously vaccinated individuals [25]. Another study from the US Military Health System showed that the timing, but not disease severity, of prior infection may predict vaccine immunogenicity [26]. The authors propose that strategies on vaccine dosing intervals should take into account the timing of SARS-CoV-2 natural infections. Furthermore, some suggested a possible association between the vaccine and the increase in excess deaths seen in Japan since 2021, when the COVID-19 vaccine was first introduced [27]. These issues question the cost-effectiveness and added benefit of booster vaccines in healthy young people.

A main limitation of this study is the use of a semi-quantitative assay that does not necessarily indicate the degree of immunity or protection from infection. Although the fold-change in the sCOVG index is lower for the fourth and fifth doses compared to the third vaccine, this could stem from higher baseline levels in the presence of a fixed upper limit of quantification. Our study did not refer to possible cases of hybrid immunity, which plays a significant role in vaccine immunogenicity. Another limitation is that the data were obtained from relatively healthy individuals, but important populations, such as the very elderly or immunocompromised, were not included. This is a significant shortcoming, since the findings obtained from a homogenous sample may not be directly generalized to the diverse global population affected by COVID-19. Lastly, our study focused solely on humoral immunity (antibody levels) and did not investigate cellular immunity, a critical component of long-term SARS-CoV-2 protection, providing a partial picture of the immune response.

## 5. Conclusions

Hopefully, a fifth COVID vaccine dose will not be put to the test in a real-word, large-scale setting. In that event, the data presented here suggest that a fifth dose elicits antibody response in healthy individuals, although the clinical and immunological implications are still unclear.

## Figures and Tables

**Table 1:** Baseline characteristics and anti-SARS-CoV-2 IgG antibody response to SARS-CoV-2 booster vaccines in 162 healthcare workers.

Total N	162		
Age, median (IQR)	69 (65–74)		
Sex, N/total (%)			
Female	78/162 (62%)		
Male	47/162 (38%)		
Comorbidities, N/total (%) *			
Hypertension	41/125 (33%)		
Dyslipidemia	58/125 (46%)		
Cancer	8/125 (6%)		
Diabetes	17/125 (14%)		
Ischemic heart disease	9/125 (7%)		
Rheumatologic disease	4/125 (3%)		
Any comorbidity	76/125 (61%)		
Vaccine Dose **	Pre-Vaccine	Post-Vaccine	Mean Fold-Change
	sCOVG index (SD)	sCOVG index (SD)	(SD)
Third dose	3.4 (2.9)	94.9 (1.3)	29.2 (2.6)
Fourth dose	34.2 (2.7)	97.8 (1.1)	3.8 (2.4)
Fifth dose	65.0 (2.6)	96.4 (1.2)	3.6 (3.0)

* Clinical data were available for 125 individuals only. ** The number of observations was 125, 118, and 58 for the third, fourth, and fifth vaccine doses, respectively.

**Table 2:** Adverse events according to a self-reported questionnaire following a fifth dose of BNT162b2 vaccine.

Total N	51 *
Early adverse events, N/total (%)	
Mild local pain	28/51 (55%)
Local erythema	8/51 (16%)
Swelling	5/51 (10%)
Movement-restricting pain	3/51 (6%)
Late adverse events (after 48 h), N/total (%)	
No symptoms	36/51 (71%)
Weakness or fatigue	9/51 (18%)
Arthralgia or myalgia	4/51 (8%)
Dizziness	3/51 (6%)
Fever	3/51 (6%)
Headache	3/51 (6%)
Symptom severity compared to previous doses, N/total (%)	
Similar degree	35/51 (69%)
Less severe	10/51 (20%)
More severe	3/51 (6%)
Do not remember	3/51 (6%)

* Safety data were available for 51 out of the 58 individuals who received a fifth vaccine dose.

## Data Availability

The datasets used during the current study are available from the corresponding author upon reasonable request.
